# Let-7a Is a Direct EWS-FLI-1 Target Implicated in Ewing's Sarcoma Development

**DOI:** 10.1371/journal.pone.0023592

**Published:** 2011-08-10

**Authors:** Claudio De Vito, Nicolo Riggi, Mario-Luca Suvà, Michalina Janiszewska, Janine Horlbeck, Karine Baumer, Paolo Provero, Ivan Stamenkovic

**Affiliations:** 1 Faculty of Biology and Medicine, Institute of Pathology, Centre Hospitalier Universitaire Vaudois, University of Lausanne, Lausanne, Switzerland; 2 Department of Biochemistry, Molecular Biology and Biotechnology, University of Torino, Torino, Italy; University of Illinois at Chicago, United States of America

## Abstract

Ewing's sarcoma family tumors (ESFT) are the second most common bone malignancy in children and young adults, characterized by unique chromosomal translocations that in 85% of cases lead to expression of the EWS-FLI-1 fusion protein. EWS-FLI-1 functions as an aberrant transcription factor that can both induce and suppress members of its target gene repertoire. We have recently demonstrated that EWS-FLI-1 can alter microRNA (miRNA) expression and that miRNA145 is a direct EWS-FLI-1 target whose suppression is implicated in ESFT development. Here, we use miRNA arrays to compare the global miRNA expression profile of human mesenchymal stem cells (MSC) and ESFT cell lines, and show that ESFT display a distinct miRNA signature that includes induction of the oncogenic miRNA 17–92 cluster and repression of the tumor suppressor let-7 family. We demonstrate that direct repression of let-7a by EWS-FLI-1 participates in the tumorigenic potential of ESFT cells in vivo. The mechanism whereby let-7a expression regulates ESFT growth is shown to be mediated by its target gene *HMGA2*, as let-7a overexpression and *HMGA2* repression both block ESFT cell tumorigenicity. Consistent with these observations, systemic delivery of synthetic let-7a into ESFT-bearing mice restored its expression in tumor cells, decreased *HMGA2* expression levels and resulted in ESFT growth inhibition in vivo. Our observations provide evidence that deregulation of let-7a target gene expression participates in ESFT development and identify let-7a as promising new therapeutic target for one of the most aggressive pediatric malignancies.

## Introduction

Transformation and subsequent cancer development require genetic events in the form of point mutation, deletion or translocation of genes that either promote or control cell growth, proliferation and survival. The effects of these genetic alterations are subject to modulation by epigenetic events whose contribution may be key to the establishment of the full fledged malignant phenotype. These include promoter methylation of tumor suppressor genes and histone modifications that regulate DNA accessibility to transcription factors. More recently, microRNAs (miRNAs) have been shown to play a major role in potentiating genetically-driven oncogenic events [1].

MicroRNAs are non-coding transcripts that undergo a defined series of processing steps, initiated by RNA polymerase II-mediated transcription to generate a primary miRNA (pri-miRNA) [1]. Pri-miRNA is processed by the multiprotein microprocessor complex that includes Drosha, an RNAseIII enzyme and DGCR8/Pasha, a double stranded RNA-binding domain protein, to produce a ∼70 nucleotide (nt) precursor miRNA (pre-miRNA). Pre-miRNA is subsequently exported by Exportin-5 from the nucleus to the cytoplasm, where it is further processed by the Dicer complex to generate the mature 21–23 nt miRNA. The two miRNA strands are then separated and the guide strand is loaded onto the the RNA-induced silencing complex (RISC) by binding to an Argonaute (Ago) protein whereas the carrier strand is degraded [1]. The miRNA guides RISC to complementary sequences within the 3̀untranslated regions (UTR), introns and even exons of a wide range of target genes. By binding to their complementary sequences, miRNAs destabilize the corresponding transcripts through mRNA degradation or reduced translation, leading to their silencing.

Because complementary sequences to a given miRNA are found in numerous genes, a restricted number of miRNAs can regulate the expression of large numbers of genes, several of which may be implicated in the control of key cell functions, including proliferation, differentiation and apoptosis [2]. Not surprisingly, changes in expression of a host of individual miRNAs have been associated with various cancer types and implicated in events ranging from transformation to cancer progression and metastasis. Because miRNAs repress target gene expression, at least two miRNA categories that are relevant to oncogenesis have been identified: those that are overexpressed in cancer cells and act as oncogenes by targeting tumor suppressive transcripts, and those that are repressed in cancer cells and act as tumor suppressor genes by targeting oncogenic transcripts [3], [4]. A mounting body of evidence suggests that malignant cells display global miRNA silencing [5]. Recent experimental evidence suggests *DICER* gene deletion in mouse models [6] and Dicer protein destabilization in human cells [7] block miRNA maturation and promote transformation and tumorigenesis. Downregulation of miRNAs has thus been associated with diverse types of cancer [1].

Recent work from our laboratory has shown that downregulation of miRNA-145 is implicated in the development of cancer stem cells (CSC) in Ewing's sarcoma family tumors (ESFT), the second most common bone malignancy in children and young adults [8]. ESFT are characterized by unique chromosomal translocations that give rise to fusion genes composed of EWS and one of several ets family members of transcription factors [9]. The most common fusion gene, *EWS-FLI-1*, arises as a result of the chromosomal translocation t(11;22)(q24;q12) and is expressed in 85–90% of ESFT [9]. The EWS-FLI-1 fusion protein is believed to provide the key oncogenic event in ESFT by inducing and repressing target genes that lead to transformation of permissive primary cells. Mesenchymal stem cells (MSC) have been shown to provide permissiveness for EWS-FLI-1 expression and oncogenicity and are currently considered to be the most likely cell of origin of ESFT [8], [10], [11], [12].

Despite the identification of their candidate cell of origin, the mechanisms that underlie ESFT formation are still incompletely understood. Although EWS-FLI-1 has the ability to directly modulate the expression of a broad repertoire of target genes, including induction and repression of oncogenes and tumor suppressor genes, respectively, these mechanisms do not provide the full explanation for ESFT pathogenesis.

Based on our recent observations that miRNA-145 repression underlies the emergence of ESFT CSC [8], we compared the miRNA expression profiles of MSCs and ESFT cell lines to identify miRNAs that may be implicated in ESFT pathogenesis and that may provide potential therapeutic targets. Our observations indicate that ESFT display concomitant induction of the oncogenic miRNA 17–92 cluster [13] and repression of the entire let-7 tumor suppressor family [14], [15]. We show the let-7 family member let-7a to be a direct EWS-FLI-1 target gene, whose *in vivo* repression promotes ESFT cell tumorigenicity via induction of its target gene *HMGA2*. More importantly, we demonstrate that systemic delivery of synthetic let-7a significantly decreases tumor growth *in vivo*, providing a potentially potent novel therapeutic option in ESFT.

## Materials and Methods

### Cell culture and retroviral infection

MSCs were isolated and cultured and infected as previously described [8]. A673 and SK-ES-1 (ATCC), TC252 (kindly provided by Dr T.Triche [8]) and STA-ET-8.2 (kindly provided by Dr H. Kovar [16]) ESFT cells lines were cultured in RPMI (Gibco, Invitrogen) supplemented with 10% FCS (Gibco) and penicillin/streptamycin (Gibco). A673 and TC252 were infected as previously described (8). Let-7a vector was kindly provided by Dr R. Agami. Informed written consent was obtained from all patients involved in the study and approval was obtained from the ethics committee of the CHUV and Faculty of Biology and Medicine of the University of Lausanne. For samples obtained from pediatric patients, written consent was provided by the parents.

### RNA isolation and real-time PCR

Total RNA was isolated using Trifast (Peqlab) according to the manufacturer's recommendations. Real time PCR was performed as previously described [8]. TaqMan probes included: Myc and Cyclophilin A. SYBR Green gene expression quantification for HMGA2 primer sequences are Fwd 5′-GCGCCTCAGAAGAGAGGAC-3′ and Rev 5′ -GTCTTCCCCTGGGTCTCTTAG-3′, IGF2BP1 primer sequences were: Fwd 5′-GGCCTGAGAATGAGTG-3′ and Rev 5′-GAGGGGCAGACAGTGTTG-3′, hnRNP A1 primer sequences were: Fwd 5′-GTCAGCTTGCTCCTTTCTGC-3′ and Rev 5′-GCTCAACCCTCCAATGAAGA-3′ Primer sequences for pri-let-7a-1, lin28A and lin28B were previously described [7] and [17]. For microRNA quantification, 30ng of total RNA were amplified using miRCURY LNA Universal RT microRNA PCR kit (Exiqon, DK) according to the manufacturer's recommendations. LNA PCR primers from Exiqon were used for RT-PCR amplification and snord49a was used as endogenous control.

### MicroRNA Array Profiling

**Figure 1:**
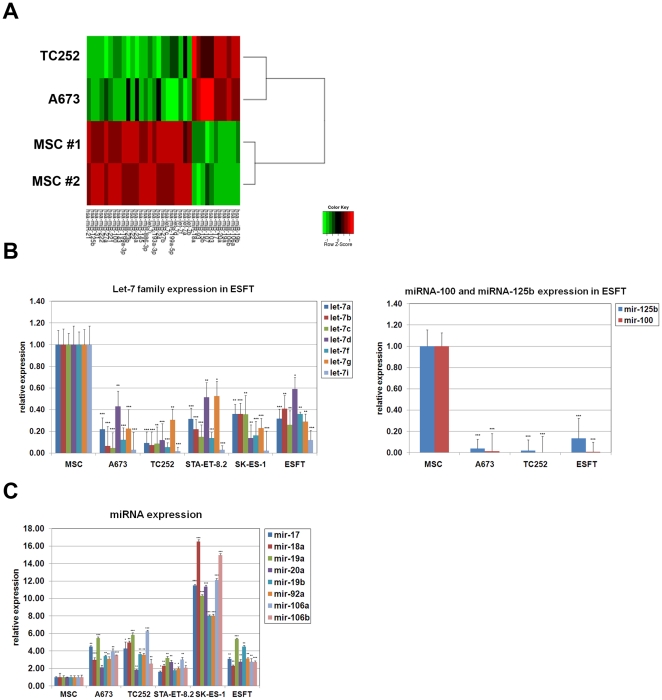
miRNAs expression in ESFT. **A**) Clustering of MSC, A673 and TC252 ESFT cell lines according to expression levels of the 35 differentially expressed microRNAs. **B**) **Left**: Real-Time PCR analysis of let-7 family expression in MSC, A673, TC252, STA-ET-8.2, SK-ES-1 cells and primary ESFT. **Right**: Real-Time PCR analysis of miRNA-100 and miRNA-125b in MSC, A673, TC252 cells and primary ESFT. **C**) Real-Time PCR analysis of miRNA-17–92 cluster and its paralogs in MSC, A673, TC252 cells and primary ESFT. Real-Time PCR experiments were normalized to SNORD49A, and done in triplicate. Error bars represent the SD of three independent experiments. Student T-test was used for statistical analysis. * p<0.05, ** p<0.005, *** p<0.0005.

RNA quality was assessed by Bioanalyzer. Sample labeling was performed according to miRXplore User manual (Miltenyi Biotech). Fluorescence signals of the hybridized miRXplore Microarrays were detected using a laser scanner from Agilent (Agilent Technologies). Signal intensity was measured using ImaGene software (Biodiscovery) and data were analyzed using the PIQOR Analyzer software. MicroRNAs displaying greater than a 2-fold change in at least 3 out of the 4 possible comparisons between the 2 ESFT cell lines and the 2 MSC samples were considered to be differentially expressed. The 35 microRNA thus selected (11 up-regulated and 24 down-regulated in ESFT cell lines compared to MSCs) were used to produce the clustering shown in Figure 1a. All data are MIAME compliant and the raw data have been deposited in a MIAME compliant database. Accession Number: GSE29085.

### Chromatin immunoprecipitation

2×10^7^ A673 cells were cross-linked with 1% PFA for 10 minutes. PFA was blocked with glycine 125 mM for 5 minutes. Cells were washed with cold PBS and then scraped on ice with PBS and proteinase inhibitor (Roche). Cells were lysated in lysis buffer (1% SDS, 10 mM EDTA, 50 mM Tris HCl pH 8.0) for 10 min and then sonicated to obtain chromatin fragments between 0.5 to 1 kb length. Cell lysate was diluted 10x in dilution buffer (1% Triton, 2 mM EDTA, 150 mM NaCl, 20 mM Tris HCl pH 8.0) and precleared 1 h with prot A sepharose beads (GE Healthcare Bio-Sciences AB). Samples were incubated overnight with either anti FLI1 antibody (Santa Cruz Biotechnology) or an unrelated isotype-matched antibody (Sigma). Protein A sepharose beads was added for 1 h at 4°C. Beads were washed 3 times with wash buffer (0.1% SDS, 1% Triton X-100, 2 mM EDTA, 150 mM NaCl, 20 mM Tris HCl pH 8.0) and once with final wash buffer (0.1% SDS, 1% Triton X-100, 2 mM EDTA, 500 mM NaCl, 20 mM Tris HCl pH 8.0). Chromatin was eluted with 1% SDS and 100 mM NaHCO_3_ for 15 min. Reverse cross-linking was performed at 65°C for 4 h and proteins were digested with proteinase K for 1 h at 45°C. Chromatin was recovered using phenol/chloroform extraction. For real time PCR the following primers were used: Fwd let-7a-1 promoter 5′ TGTGGCTATACAGCCGTCAG 3′ and Rev let-7a-1 promoter 5′ACCCATACATGGGTCAACTG 3′. For non-specific binding region previously published primer sequences were used [18].

### Luciferase assay

Let-7a-1 promoter was amplified from genomic DNA from hpMSC and cloned into pGL3 luciferase vector (Promega) using the following primer sequences: Fwd KpnI 5′-GGGGTACCCCTGTGTGTTTTGCACACCAGTTTAC-3′ and Rev NheI 5′ –CTAGCTAGCTAGAGTGAAGAGAACATCCAGGGTGAATG-3′. HEK-293T cells were co-transfected with empty pGL3 or let-7a-1 promoter pGL3 vector along with pMSCV empty or pMSCV EWS-FLI-1-V5 vector and renilla vector using Fugene (Roche). 48 h after transfection luciferase and renilla activity were measured using Dual-lucifease Reporter assay system (Promega) according to manufacturer's instructions.

### HMGA2 construct, HMGA2 and EWS-FLI-1 depletion

HMGA2 was amplified from A673 cDNA and cloned into pMSCV puro vector (Clonetech) using the following primer sequences: Fwd BglII 5′- GAAGATCTTCCCACCATGAGCGCACGCGGTGAGGG-3′ and Rev EcoRI 5′- GGAATTCCTCAGTGACTCCTTTTGGTACTGTTTC-3′.Sequences #1 and #2 for stable knock-down of HMGA2 are respectively: 5′- CGGCCAAGAGGCAGACCTA - 3′ and 5′- GCGCCAACGTTCGATTTCT - 3′. The sequence for stable EWS-FLI-1 knock-down was previously described [12]. Oligonucleotides were annealed and cloned in pSiren RetroQ vector (Clontech).

### Western blot analysis

Western blot was performed as previously described [8]. Antibodies used for the study were anti-: c-Myc (Santa Cruz Biotechnology), HMGA2 (Biocheck, Inc. Foster City, CA), IGF2BP1 (Cell Signaling), Lin28B (Cell Signaling), Fli-1 (Santa Cruz Biotechnology) and β-actin (Sigma). Densitometric quantification was performed using Image J software.

### Tumorigenicity assay

NOD-SCID mice were anesthetized and 1×10^6^ or 2×10^6^ A673 or TC252 cells respectively were injected subcutaneously. The animals were sacrificed 4 weeks after injection. All tumors were resected at autopsy and sectioned for histological analysis. For *in vivo* treatment assay, 30 µg of miScript microRNA mimic (Qiagen) was formulated with MaxSuppressor *in vivo* RNALancerII (BIOO Scientific, Inc), according to the manufacturer recommendations. MiRNAs were administrated intravenously by tail vein injection at day 11, 14, and 17. Tumor volume was measured as previously described [19]. Experimental protocols involving mice were approved by the Etat de Vaud, Service Vétérinaire, authorization number VD1477.2.

### MTT assay

MTT assays were performed according to standard procedures.

### Immunohistochemistry

Paraffin-embedded sections of primary Ewing's sarcoma, TC252- and A673-derived tumors were stained with rabbit anti-human HMGA2 polyclonal antibody (Biocheck) or rabbit anti-human Lin28B polyclonal antibody (Cell Signaling). Horseradish peroxidase staining was performed using biotin conjugated goat anti-rabbit (Vector Laboratories), and was revealed with a DAKO DAB Kit (Dako).

## Results

### Expression profile comparison of ESFT cell lines to MSC identify a restricted cluster of differentially expressed miRNAs in ESFT

To identify miRNAs that may be involved in ESFT development we performed miRNA expression profiling of MSCs and two ESFT cell lines (A673 and TC252). A673 and TC252 were chosen because they bear a different p53 status, the latter having been shown to influence miRNA maturation [20]. A673 cells harbor mutated p53 whereas TC252 cells express the wt form [21]. The two ESFT cell lines revealed a similar miRNA expression profile, characterized by repression of the entire let-7 family, miRNA-100, miRNA-125b and miRNA-31, and over-expression of the miRNA 17–92 cluster and its paralogs miRNA-106a and miRNA-106b (Figure 1A). The expression status of these miRNAs was validated by qRT-PCR in 3 primary ESFT and four ESFT cell lines (A673, TC252, STA-ET-8.2 and SK-ES-1, Figure 1B, C and Figure S1A). The observed changes in miRNA expression are consistent with reports that members of the let-7 family are repressed in a broad range of tumors [14], that miRNA-31 is repressed in disseminating and metastatic malignancies [22], [23] and that overexpression of the cluster 17–92 is involved in the tumorigenic phenotype of haematologic malignancies as well as that of a variety of solid tumors [13].

### Let-7a is a direct EWS-FLI-1 target implicated in ESFT cell tumorigenicity

**Figure 2:**
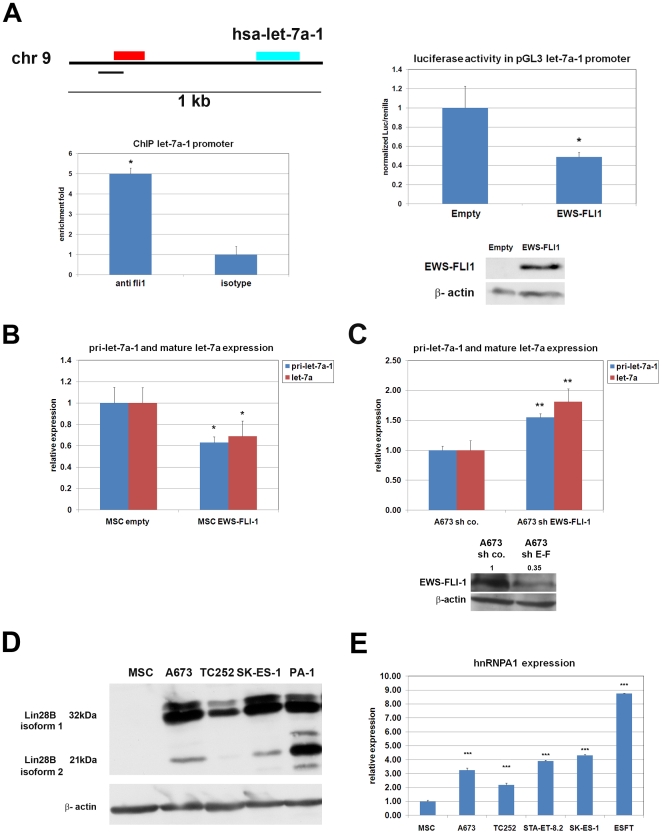
let-7a is a direct target of EWS-FLI-1. **A**) **Left**: Upper panel: 1 kb genomic region encompassing the let-7a-1 locus. Blue bar: genomic region coding for the precursor let-7a-1, red bar: genomic region containing the putative let-7a-1 promoter, as identified by Gangwal and al [27]. Lower panel: Real-Time PCR analysis of anti-Fli-1 or isotype-matched control antibody immunoprecipitated chromatin in A673 cells showing a 5-fold enrichment of the let-7a-1 promoter with anti-Fli-1 antibody. **Right**: let-7a-1 promoter luciferase assay showing EWS-FLI-1-mediated repression of luciferase activity in HEK-293T cells (upper panel), and expression of the EWS-FLI-1 fusion protein in the corresponding cell population (lower panel). **B**) Real-Time PCR analysis of pri-let-7a-1 and mature let-7a expression in human pediatric MSC^EWS-FLI-1^. **C**) Real-Time PCR analysis of pri-let-7a-1 and mature let-7a in EWS-FLI-1-depleted A673. **D**) Western blot analysis of *LIN28B* expression in MSC, ESFT cell lines and PA-1 teratocarcinoma cell line. **E**) Real-Time PCR analysis of hnRNP A1 expression in MSC, ESFT cell lines and primary ESFT. Real-Time PCR experiments were normalized to Cyclophilin A, and done in triplicate. Error bars represent the SD of three independent experiments. Student T-test was used for statistical analysis.* p<0.05, ** p<0.005, *** p<0.0005.

Members of the let-7 family play a major role in cell differentiation and are considered to act as tumor suppressors by silencing numerous genes that encode oncogenic proteins including *HMGA2*, insulin-like growth factor 2-binding protein 1 (*IGF2BP1*), *RAS* and c-*MYC* [24], [25]. Although low expression of all the let-7 family members was observed in primary ESFT cells and ESFT cell lines compared to MSCs (Figure 1A), we focused our attention on let-7a because its repression has been shown to be directly implicated in the pathogenesis of several cancer types as well as in maintenance of breast cancer CSC [15], [26]. Moreover, despite the ability of EWS-FLI-1 to directly induce expression of c-Myc, a known regulator of let-7 expression, recent evidence suggests that let-7a may also constitute a direct EWS-FLI-1 target gene [27], further supporting possible involvement of let-7a in ESFT development. To verify this notion, we performed chromatin immunoprecipitation (ChIP) in A673 cells using anti-Fli-1 antibody and observed a five-fold enrichment of the let-7a-1 promoter region, compared to the isotype-matched control antibody, as assessed by Real-Time PCR analysis, indicating that EWS-FLI-1 directly binds to the let-7a-1 promoter (Figure 2A, left). To determine the functional consequence of the observed binding we engineered a luciferase reporter vector that expresses the luciferase gene under the control of the let-7a-1 promoter. Luciferase activity was reduced by 50% upon EWS-FLI-1 expression in HEK-293T cells, indicating that EWS-FLI-1 is able to repress let-7a-1 promoter activity *in vivo* (Figure 2A, right). To further verify the ability of EWS-FLI-1 to repress let-7a at the transcriptional level, we introduced EWS-FLI-1 into human pediatric MSC cultured in serum free medium by retroviral infection as previously described (8). Expression of EWS-FLI-1 resulted in reduction of pri-let-7a-1 and mature let-7a expression (Figure 2B). Real-Time PCR comparison of the pri-let-7a-1 transcript in MSCs, ESFT cell lines and primary ESFT revealed decreased expression in all ESFT cell lines tested as well as in primary ESFT (Figure S1B), supporting the notion that EWS-FLI-1 directly represses let-7a promoter activity *in vivo*. To further validate the observed repression of the let-7a-1 promoter by EWS-FLI-1, we silenced EWS-FLI-1 in A673 using an shRNA approach. Five days following EWS-FLI-1 silencing upregulation of the pri-let-7a-1 transcript as well as mature let-7a was recorded (Figure 2C). These observations explain, at least in part, the low let-7a transcript levels observed in Ewing's sarcoma cells.

Expression of the let-7 family is also known to be tightly regulated at the post-transcriptional level by *LIN28*, a direct let-7 target gene [28], [29], [30]. Lin28 is an RNA-binding protein highly expressed in embryonic stem and progenitor cells as well as in a wide range of human cancers but not in most adult tissues. Lin28 is strongly implicated in the induction and control of pluripotency and binds to the terminal loops of let-7 precursors, thereby blocking their processing to mature forms [30]. To determine whether lin28 may also participate in the regulation of let-7 expression in ESFT, we compared the expression levels of *LIN28A* and *LIN28B* in MSC, ESFT cell lines and primary tumors. No significant difference in *LIN28A* expression was observed (data not shown), whereas *LIN28B* was more highly expressed in ESFT cell lines than in MSC (Figure 2D). Recently hnRNP A1 has been shown to bind to the terminal loop of pri-let-7a-1 and impair its maturation [31]. To determine whether hnRNP A1 might participate in the blockade of pri-let-7a-1 maturation, we measured by Real-Time PCR the expression level of hnRNP A1 in MSC, ESFT cell lines and primary ESFT. Higher expression of hnRNP A1 in ESFT cell lines and primary tumors was observed compared to MSC (Figure 2E). Taken together these results suggest that in ESFT let-7a is subject to a triple repressive regulatory mechanism at both transcriptional and post-transcriptional levels as a result of direct binding of EWS-FLI-1 to its promoter and lin28B-hnRNP A1-mediated maturation blockade, respectively.

**Figure 3:**
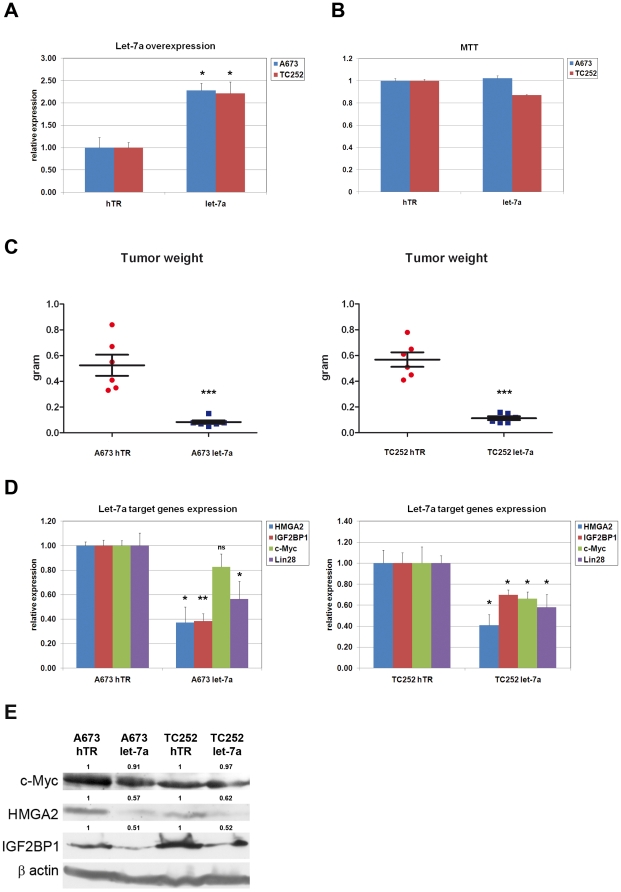
let-7a blocks ESFT tumor growth. **A**) **Left**: Real-Time PCR analysis of let-7a overexpression in the A673 and TC252 ESFT cell lines. **B**) MTT proliferation assay of mock-infected and let-7a-overexpressing A673 and TC252 cells. **C**) let-7a overexpression in A673 (left panel) and TC252 (right panel) tumor cells leads to a significant decrease in their tumorigenicity. **D**) Real-Time PCR analysis of let-7a target genes *HMGA2, IGF2BP1*, *c-MYC* and *LIN28B* expression in mock-infected and let-7a-overexpressing A673 and TC252 cells. **E**) Western Blot analysis showing reduction of HMGA2 and IGF2BP1 expression of in let-7a overexpressing A673 and TC252 cells. Real-Time PCR experiments were normalized to either Cyclophilin A for mRNA or SNORD49A for miRNAs, and done in triplicate. Error bars represent the SD of three independent determinations. Student T-test was used for statistical analysis.* p<0.05, ** p<0.005, *** p<0.0005.

Because let-7a has been widely described as a tumor suppressor miRNA, we investigated its putative role in ESFT development. To this end, let-7a was overexpressed in the two ESFT cell lines (A673 and TC252) using a retroviral system, which resulted in a 2.5 fold increase in its expression (Figure 3A). Cell proliferation was almost unaffected, as assessed by MTT assay (Figure 3B). Six NOD/SCID mice were then injected subcutaneously with 1×10^6^ or 2×10^6^ let-7a-expressing or empty vector-infected A673 and TC252 cells, respectively, and tumor formation was assessed weekly for four weeks, whereupon the animals were sacrificed. Although all mice developed tumors, even moderate let-7a overexpression resulted in a marked decrease in tumor volume irrespective of their cell line derivation (Figure 3C), consistent with findings in other tumor types, where let-7 expression was reported to delay tumor growth [19]. These observations indicate that direct repression of the let-7a promoter activity exerted by EWS-FLI-1 contributes to ESFT development. The discrepancy between the lack of any significant effect of let-7a expression on proliferation and its potent inhibition of tumor growth is not surprising, as decreased stemness coupled to reduced tumor initiating potential that should result from let7a expression may be independent of cell division.

### The let-7a target gene HMGA2 promotes tumorigenicity in ESFT cells

**Figure 4:**
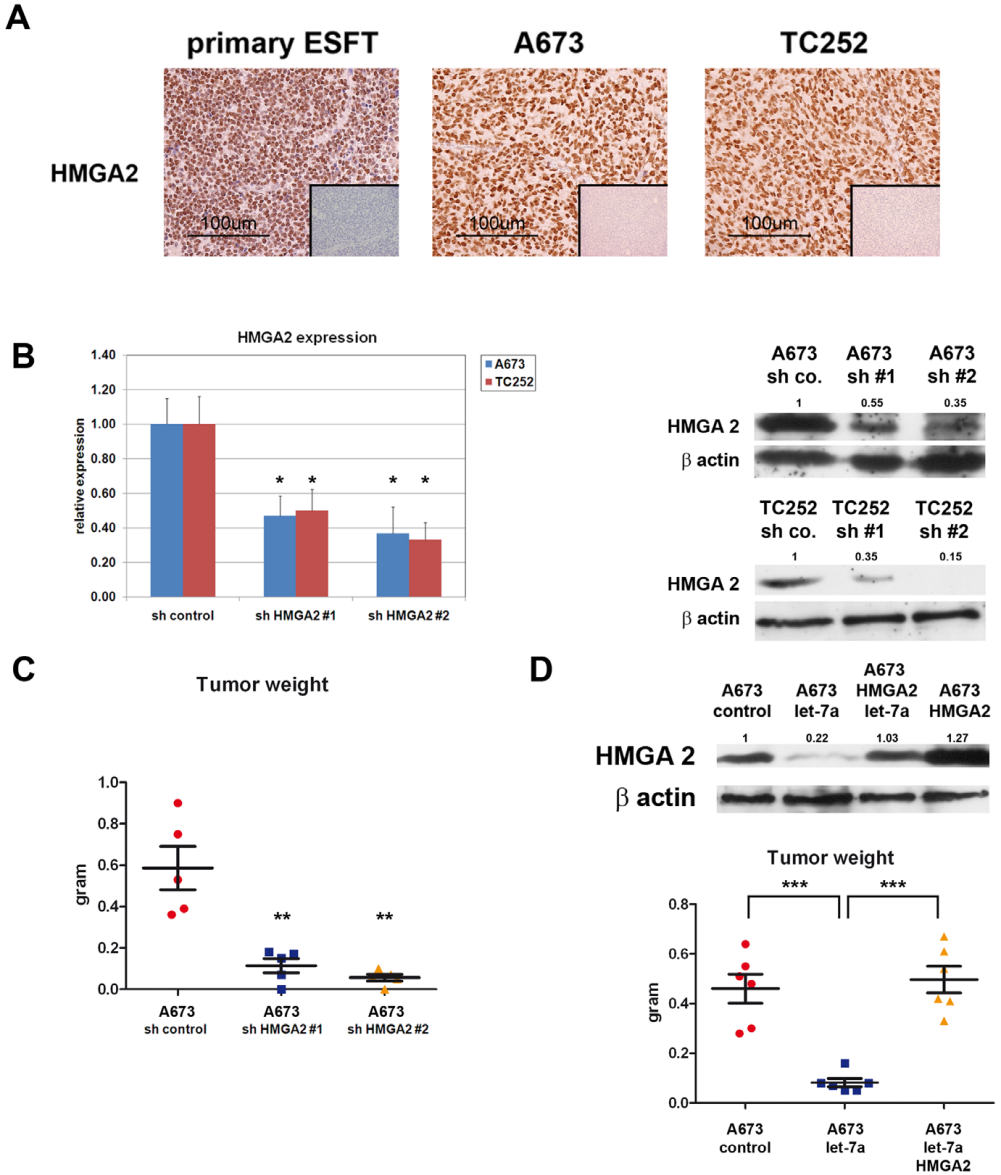
let-7a overexpression-associated loss of tumorigenicity is HMGA2-dependent. **A**) Immunohistochemical assessment of HMGA2 expression in primary ESFT and A673 and TC252 cell line-derived tumors grown in immunocompromised mice. Magnification 100x. **B**) Real-Time PCR (left panel) and Western blot analysis (right panel) of HMGA2 depletion in A673 and TC252 cells. **C**) Depletion of HMGA2 in A673 cells reduces corresponding tumor growth. **D**) **Upper panel**: Western blot analysis of HMGA2 expression in A673 cells transduced with empty vector, let-7a alone, let-7a and HMGA2 or HMGA2 alone. **Lower panel**: HMGA2 expression in A673 cells overexpressing let-7a rescues their tumorigenic properties. Real-Time PCR experiments were normalized to Cyclophilin A, and done in triplicate. Error bars represent the SD of three independent experiments. Student T-test was used for statistical analysis.* p<0.05, ** p<0.005, *** p<0.0005.

To gain insight into the mechanism whereby let-7a over-expression inhibits ESFT growth, expression levels of a panel of established let-7a target genes known to display oncogenic properties, including *HMGA2, IGF2BP1*, c-*MYC* and *LIN28B*, were assessed by Real-Time PCR in empty-vector infected and let-7a-transduced A673 and TC252 cells (Figure 3D). Significant reduction in *HMGA2, IGF2BP1* and *LIN28B* expression at both the mRNA and protein levels was observed in both cell lines upon let-7a over-expression, whereas *c-MYC* expression was mildly affected in TC252 but not A673 cells (Figure 3D, 3E and data not shown). Among the genes that we found to be repressed upon let-7a expression in ESFT cell lines, we focused on *HMGA2* because of its recognized role as an oncogene in a variety of human tumors [32]. HMGA2 is a DNA binding protein highly expressed in embryonic stem cells and cancer cells but undetectable in most normal adult tissues. It functions to alter chromatin structure in order to regulate expression of its target gene repertoire by enhancing or suppressing the activity of a variety of transcription factors [32]. HMGA2 has been found to be frequently expressed in benign mesenchymal tumors, including lipomas and leiomyomas, and to be amplified or rearranged in both well and poorly differentiated liposarcomas [33]. Immunohistochemical analysis showed strong nuclear HMGA2 expression in primary ESFT and both A673 and TC252 cell line-derived tumors (Figure 4A). To determine whether the reduction in tumor growth displayed by let-7a-expressing tumor cells may be at least in part mediated by the concomitant decrease in HMGA2 expression, we used an shRNA approach to deplete HMGA2 expression in A673 and TC252 cells. Retroviral vectors expressing two independent shRNA sequences targeting *HMGA2* were used to infect A673 and TC252 cells, and a vector containing an unrelated shRNA sequence was used as control. The resulting *HMGA2* knock-down efficiency of about 70% was similar to the effect of let-7a overexpression in the same cells, as assessed by Real-Time PCR and Western Blot analysis (Figure 4B). Depletion of HMGA2 minimally affected cell proliferation, as assessed by MTT assays (Figure S1C left). However, subcutaneous injection of HMGA2-depleted A673 and TC252 cells into NOD-SCID mice resulted in markedly reduced tumor growth, mimicking the effect of let-7a overexpression (Figure 4C and Figure S1C right). To verify that the reduction of tumor growth associated with let-7a expression was HMGA2-dependent, we performed a rescue experiment where the cDNA encoding HMGA2 lacking its endogenous 3′ UTR was expressed in A673 and TC252 cells prior to their infection with the let-7a-expressing retrovirus. Because of the lack of the 3′ UTR that contains the let-7a binding sites, exogenous cDNA-mediated expression of HMGA2 should remain unaffected by let-7a expression whereas endogenous transcripts should be silenced. Real-Time PCR and Western Blot analysis confirmed that HMGA2-infected A673 and TC252 cells expressed higher HMGA2 transcript and protein levels than empty vector infected cells (Figure 4D and data not shown). Importantly, however, HMGA2 expression in these cells returned to the pre-infection wild-type baseline level upon let-7a expression, consistent with selective silencing of the endogenous transcripts (Figure 4D and data not shown). A673 and TC252 cells expressing HMGA2 alone, let-7a alone or the combination of the two genes were injected subcutaneously into NOD/SCID mice, and the tumor forming ability of the different cell populations compared to that of their empty vector-infected counterparts. HMGA2-expressing cells failed to show any significant difference in tumor forming capacity compared to control cells. By contrast, let-7a only expressing cells displayed decreased tumorigenicity that was restored in cells expressing both HMGA2 and let-7a transcripts (Figure 4D and Figure S1D). To confirm that expression of HMGA2 was altered in let-7a expressing cell-derived tumors we assessed its expression by immunohistochemical staining in ESFT cell line-derived tumors. Consistent with the notion that HMGA2 is a target of let-7a expression of HMGA2 was decreased in tumors derived from let-7a expressing ESFT cells (Figure S2A). These observations uncover the important effector role of HMGA2 in ESFT cell tumorigenicity and identify let-7a-mediated repression of HMGA2 as a key mechanism in the reduction of tumor forming capacity by tumor cells upon let-7a restoration.

### Synthetic let-7a decreases ESFT growth in vivo

**Figure 5:**
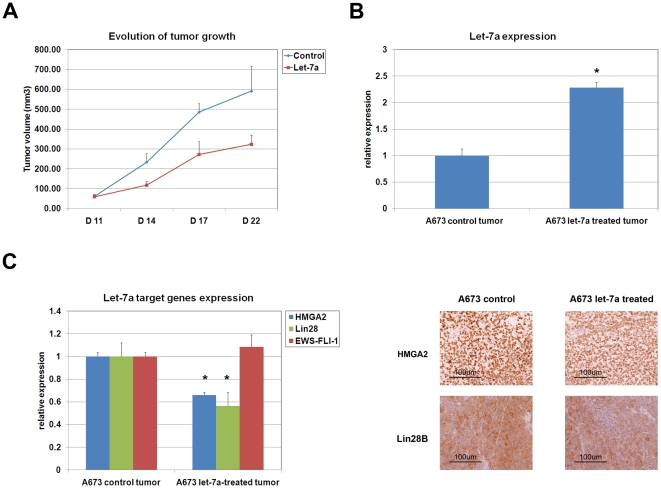
Systemic let-7a delivery reduces ESFT tumor growth in vivo. **A**) Tail vein injection of 30 µg of synthetic let-7a reduces the growth of established ESFT tumors. Let-7a was administered on days 11, 14 and 17 following the initial subcutaneous injection of A673 tumor cells. Mice were sacrificed on day 22 and tumor size assessed. **B**) Real-Time PCR analysis of let-7a expression in A673-treated or control tumors. **C**) **Left**: Real-Time PCR analysis of *HMGA2* and *LIN28B* expression in A673-treated or control tumors. **Right**: immunohistochemical staining of HMGA2 and Lin28B in A673-treated or control tumors. Magnification 100x. Real-Time PCR experiments were normalized to SNORD49a or Cyclophilin A, and done in triplicate. Error bars represent the SD of three independent determinations. Student T-test was used for statistical analysis.* p<0.05.

Our observations thus far prompted us to assess the effectiveness of administering let-7a to block ESFT growth. Feasibility of miRNA-based therapy has been demonstrated for both primary[34] and metastatic tumor growth [35]. A variety of approaches have been used to administer miRNA, including intra-tumoral and intra-venous injection. We selected to deliver miRNAs into the bloodstream of mice bearing tumors because it corresponds to the principal mode of chemotherapeutic drug administration. A673 cells were injected subcutaneously into NOD-SCID mice and let-7a was administered once tumors reached a volume of 60 mm^3^. 30 µg of synthetic miRNA, corresponding to a concentration of 1.5 µg/g, were injected into the tail vein in a single bolus on days 11, 14 and 17 post tumor cell grafting and tumor volume was measured every three days starting from the first injection. Marked reduction of tumor growth in mice treated with synthetic let-7a was observed (Figure 5A). To verify that the observed reduction of tumor growth was the consequence of exogenous miRNA administration we performed Real-Time PCR comparison of let-7a expression between treated and control tumors. Let-7a expression was found to be increased in treated tumors compared to their control counterparts, confirming that the injected synthetic miRNAs had reached the developing tumors (Figure 5B). To formally demonstrate that the delivered miRNA had been incorporated into the target tumor cells, we assessed the expression of *HMGA2* and *LIN28B* by Real-Time PCR, immunohistochemical staining and Western blot analysis and observed a decrease in both transcript and protein levels in let-7a-treated tumors (Figure 5C and Figure S2B). Heterogeneous expression of HMGA2 and lin28B was observed in let-7a-treated tumors suggesting non uniform distribution of synthetic miRNA within the tumor.

## Discussion

We have previously identified miRNA-145 as a direct EWS-FLI-1 target gene, whose repression is implicated in ESFT development, suggesting that other miRNAs may be involved in the pathogenesis of these tumors. Using miRNA array profiling we uncovered a limited number of differentially expressed miRNA families in ESFT cells. Among induced miRNAs, we found the oncogenic miRNA 17–92 cluster and its paralogs miRNA106a/b, whereas repressed miRNAs included miRNA 100, 125b as well as the entire let-7 family. Interestingly, the miRNA 17–92 cluster has been reported to be directly induced by c-Myc, a known EWS-FLI-1 target gene [36], suggesting that this cluster may be indirectly modulated by EWS-FLI-1 through c-Myc induction. Among the let-7 miRNA family we focused on let-7a because of its reported functional role in diverse cancer types.

### Let-7a repression and the corresponding upregulation of its target gene HMGA2 provide a new mechanism of sustained ESFT growth

Let-7a repression has been observed in diverse malignant tumor types, including a variety of sarcomas and carcinomas. Let-7a down-regulation is mediated by several mechanisms including Lin28-dependent degradation (27–29) and myc-dependent transcriptional repression [37]. In ESFT, we have shown that direct EWS-FLI-1-mediated repression provides a novel regulatory mechanism of let-7a expression. Given its role as an inhibitor of differentiation [38], let-7a repression may participate in early EWS-FLI-1-mediated transformation, by enhancing primary cell permissiveness for EWS-FLI-1 expression and function, as well as in subsequent ESFT CSC maintenance. The observed upregulation of the stem cell gene *LIN28B* in ESFT is consistent with its reported function as an oncogene whose expression in human cancer is associated with unfavorable prognosis [17]. LIN28 is an RNA binding protein whose expression is normally restricted to embryonic stem and progenitor cells as well as developing tissues, where it plays the role of a master regulator of pluripotency. Together with OCT-4, SOX2 and NANOG, LIN28 is also involved in genetic reprogramming that leads to generation of induced pluripotent stem cells (iPS) *in vitro* [39]. Reactivation of its expression may therefore constitute one of the mechanisms that links genetic reprogramming to CSC emergence in human tumors. LIN28 has recently been shown to be directly involved in the generation and maintenance of ovarian aldehyde dehydrogenase (ALDH)-positive CSC through its ability to block let-7 maturation [40]. Together with increasing evidence of a pivotal role of let-7 in normal and cancer stem cell differentiation, this observation further supports the notion that the double negative feedback loop between LIN28 and let-7 may regulate the behavior of CSC *in vivo*. In the context of the recent report that ESFT CSC express high ALDH levels [41], it is tempting to speculate that let-7a and miRNA-145 repression may play a critical role in EWS-FLI-1-mediated CSC generation. Similar to our discovery that repression of miRNA-145 is directly involved in the emergence of ESFT CSC [8], the observed repression of let-7a may enhance expression of *LIN28B*, triggering a double negative feed-back loop that reinforces let-7 repression in ESFT and facilitates CSC generation and maintenance.

Let-7a target genes relevant to transformation and subsequent tumor development include *RAS, MYC, IGF2BP1* and *HMGA*2. HMGA2 is of particular interest in ESFT because its overexpression is associated with both benign and malignant mesenchymal tumors [42] and because it has been shown to regulate mesenchymal stem cell genes [43]. HMGA2 is a DNA binding protein that does not have transcriptional activity but rather cooperates with the transcription machinery to alter chromatin structure, thereby inducing or silencing numerous genes. Among its known functions are induction of E2F activity, cyclin A and pro-inflammatory protein expression, modulation of miRNA expression as well as genes implicated in epithelial to mesenchymal transition and inhibition of p53-mediated apoptosis [32]. In ESFT cells, HMGA2 depletion resulted in markedly reduced tumor growth, consistent with a role in CSC maintenance by a variety of possible mechanisms that include inhibition of the oncogenic stress response to EWS-FLI-1, promotion of stemness as a consequence of chromatin modification and maintenance of cell cycle. In support of this notion, HMGA2 has been recently reported to participate in self-renewal of neural stem cells by controlling the INK4A locus [44]. Thus by virtue of its selective overexpression in tumor cells and likely role in CSC maintenance, HMGA2 may constitute a therapeutic target of interest.

### Targeting of selected miRNA provides a means to control ESFT growth

Reversion of miRNA suppression mechanisms in CSC could conceptually lead to abrogation of their stem cell properties and elimination of their tumor repopulating capacity. However, this would require in depth understanding of the mechanisms involved, which, as is increasingly apparent, may be multiple with uncertain targetability [1], [7]. An alternative approach is to restore relevant miRNA expression by systemic administration of synthetic miRNA *in vivo*. Synthetic miRNAs have the advantage of being easy to engineer and of being stable. More importantly, miRNA administration may be devoid of major secondary effects as differentiated cells already express high miRNA levels to which administration of exogenous species is unlikely to contribute in significant fashion. Thus, exogenous miRNA administration can selectively replenish cells that display inappropriate miRNA repression associated with disease. Our observations demonstrate the feasibility of reducing ESFT growth *in vivo* by administering relatively low doses of synthetic let-7a. Moreover, they provide evidence of miRNA delivery to the appropriate tumor target cells and their effect within the cells as illustrated by the expected alteration in target gene expression levels.

Taken together, our observations have identified a miRNA expression signature that characterizes ESFT and that participates in ESFT pathogenesis, including the miRNA tumor suppressor family let-7. We have also shown that EWS-FLI-1 directly binds to the let-7a promoter, repressing its transcriptional activity, and that reduced let-7a expression is implicated in ESFT development through HMGA2 regulation. Finally, restoration of let-7a expression by an approach as simple as *in vivo* systemic delivery of synthetic miRNAs may provide the means to control malignancies as aggressive as ESFT.

## Supporting Information

Figure S1 **miRNA expression in STA-ET-8.2 and SK-ES-1 ESFT cell lines, MTT and tumorigenic assay of HMGA2-depleted TC252 tumor cells.** **A**) Real-Time PCR analysis of miRNA-100, miRNA-125b in MSC, STA-ET-8.2 and SK-ES-1 cells. **B**) Real-Time PCR analysis of pri-let-7a-1 expression in MSC, ESFT cell lines and primary ESFT. **C**) **Left**: MTT assay of mock-infected and HMGA2-depleted ESFT tumor cells. **Right**: Depletion of HMGA2 in TC252 cells reduces corresponding tumor growth. **D**) HMGA2 expression in TC252 cells overexpressing let-7a rescues their tumorigenic properties. Error bars represent the SD of three independent determinations. Student T-test was used for statistical analysis, ** p<0.005, *** p<0.0005.
(TIF)

Figure S2 **HMGA2 and Lin28 expression in let-7a and let-7a-HMGA2 expressing ESFT cells derived tumors.** **A**) Immunohistochemical staining of HMGA2 in mock- let-7a and let-7a-HMGA2 expressing ESFT cell lines derived tumors. **B**) Western blot analysis of tumors from mice treated with let7a and vehicle only. Magnification 100x.
(TIF)

## References

[1] Melo SA, Esteller M (2011). Dysregulation of microRNAs in cancer: Playing with fire.. FEBS Lett.

[2] Bartel DP (2009). MicroRNAs: target recognition and regulatory functions.. Cell.

[3] Ventura A, Jacks T (2009). MicroRNAs and cancer: short RNAs go a long way.. Cell.

[4] Plasterk RH (2006). Micro RNAs in animal development.. Cell.

[5] Lu J, Getz G, Miska EA, Alvarez-Saavedra E, Lamb J (2005). MicroRNA expression profiles classify human cancers.. Nature.

[6] Kumar MS, Lu J, Mercer KL, Golub TR, Jacks T (2007). Impaired microRNA processing enhances cellular transformation and tumorigenesis.. Nat Genet.

[7] Melo SA, Ropero S, Moutinho C, Aaltonen LA, Yamamoto H (2009). A TARBP2 mutation in human cancer impairs microRNA processing and DICER1 function.. Nat Genet.

[8] Riggi N, Suva ML, De Vito C, Provero P, Stehle JC (2010). EWS-FLI-1 modulates miRNA145 and SOX2 expression to initiate mesenchymal stem cell reprogramming toward Ewing sarcoma cancer stem cells.. Genes Dev.

[9] Riggi N, Stamenkovic I (2007). The Biology of Ewing sarcoma.. Cancer Lett.

[10] Riggi N, Cironi L, Provero P, Suva ML, Kaloulis K (2005). Development of Ewing's sarcoma from primary bone marrow-derived mesenchymal progenitor cells.. Cancer Res.

[11] Riggi N, Suva ML, Suva D, Cironi L, Provero P (2008). EWS-FLI-1 expression triggers a Ewing's sarcoma initiation program in primary human mesenchymal stem cells.. Cancer Res.

[12] Tirode F, Laud-Duval K, Prieur A, Delorme B, Charbord P (2007). Mesenchymal stem cell features of ewing tumors.. Cancer Cell.

[13] Mendell JT (2008). miRiad roles for the miR-17-92 cluster in development and disease.. Cell.

[14] Bussing I, Slack FJ, Grosshans H (2008). let-7 microRNAs in development, stem cells and cancer.. Trends Mol Med.

[15] Yu F, Yao H, Zhu P, Zhang X, Pan Q (2007). let-7 regulates self renewal and tumorigenicity of breast cancer cells.. Cell.

[16] Jiang X, Gwye Y, Russell D, Cao C, Douglas D (2010). CD133 expression in chemo-resistant Ewing sarcoma cells.. BMC Cancer.

[17] Viswanathan SR, Powers JT, Einhorn W, Hoshida Y, Ng TL (2009). Lin28 promotes transformation and is associated with advanced human malignancies.. Nat Genet.

[18] Richter GH, Plehm S, Fasan A, Rossler S, Unland R (2009). EZH2 is a mediator of EWS/FLI1 driven tumor growth and metastasis blocking endothelial and neuro-ectodermal differentiation.. Proc Natl Acad Sci U S A.

[19] Esquela-Kerscher A, Trang P, Wiggins JF, Patrawala L, Cheng A (2008). The let-7 microRNA reduces tumor growth in mouse models of lung cancer.. Cell Cycle.

[20] Suzuki HI, Yamagata K, Sugimoto K, Iwamoto T, Kato S (2009). Modulation of microRNA processing by p53.. Nature.

[21] Kovar H, Jug G, Aryee DN, Zoubek A, Ambros P (1997). Among genes involved in the RB dependent cell cycle regulatory cascade, the p16 tumor suppressor gene is frequently lost in the Ewing family of tumors.. Oncogene.

[22] Valastyan S, Weinberg RA (2010). miR-31: A crucial overseer of tumor metastasis and other emerging roles.. Cell Cycle.

[23] Valastyan S, Reinhardt F, Benaich N, Calogrias D, Szasz AM (2009). A pleiotropically acting microRNA, miR-31, inhibits breast cancer metastasis.. Cell.

[24] Boyerinas B, Park SM, Shomron N, Hedegaard MM, Vinther J (2008). Identification of let-7-regulated oncofetal genes.. Cancer Res.

[25] Kumar MS, Erkeland SJ, Pester RE, Chen CY, Ebert MS (2008). Suppression of non-small cell lung tumor development by the let-7 microRNA family.. Proc Natl Acad Sci U S A.

[26] Boyerinas B, Park SM, Hau A, Murmann AE, Peter ME (2010). The role of let-7 in cell differentiation and cancer.. Endocr Relat Cancer.

[27] Gangwal K, Sankar S, Hollenhorst PC, Kinsey M, Haroldsen SC (2008). Microsatellites as EWS/FLI response elements in Ewing's sarcoma.. Proc Natl Acad Sci U S A.

[28] Newman MA, Thomson JM, Hammond SM (2008). Lin-28 interaction with the Let-7 precursor loop mediates regulated microRNA processing.. RNA.

[29] Viswanathan SR, Daley GQ, Gregory RI (2008). Selective blockade of microRNA processing by Lin28.. Science.

[30] Heo I, Joo C, Cho J, Ha M, Han J (2008). Lin28 mediates the terminal uridylation of let-7 precursor MicroRNA.. Mol Cell.

[31] Michlewski G, Caceres JF (2010). Antagonistic role of hnRNP A1 and KSRP in the regulation of let-7a biogenesis.. Nature structural & molecular biology.

[32] Fusco A, Fedele M (2007). Roles of HMGA proteins in cancer.. Nat Rev Cancer.

[33] Italiano A, Bianchini L, Keslair F, Bonnafous S, Cardot-Leccia N (2008). HMGA2 is the partner of MDM2 in well-differentiated and dedifferentiated liposarcomas whereas CDK4 belongs to a distinct inconsistent amplicon.. Int J Cancer.

[34] Wiggins JF, Ruffino L, Kelnar K, Omotola M, Patrawala L (2010). Development of a lung cancer therapeutic based on the tumor suppressor microRNA-34.. Cancer Res.

[35] Ma L, Reinhardt F, Pan E, Soutschek J, Bhat B (2010). Therapeutic silencing of miR-10b inhibits metastasis in a mouse mammary tumor model.. Nat Biotechnol.

[36] O'Donnell KA, Wentzel EA, Zeller KI, Dang CV, Mendell JT (2005). c-Myc-regulated microRNAs modulate E2F1 expression.. Nature.

[37] Chang TC, Yu D, Lee YS, Wentzel EA, Arking DE (2008). Widespread microRNA repression by Myc contributes to tumorigenesis.. Nat Genet.

[38] Droge P, Davey CA (2008). Do cells let-7 determine stemness?. Cell Stem Cell.

[39] Yu J, Vodyanik MA, Smuga-Otto K, Antosiewicz-Bourget J, Frane JL (2007). Induced pluripotent stem cell lines derived from human somatic cells.. Science.

[40] Yang X, Lin X, Zhong X, Kaur S, Li N (2010). Double-Negative Feedback Loop between Reprogramming Factor LIN28 and microRNA let-7 Regulates Aldehyde Dehydrogenase 1-Positive Cancer Stem Cells.. Cancer Res.

[41] Awad O, Yustein JT, Shah P, Gul N, Katuri V (2010). High ALDH Activity Identifies Chemotherapy-Resistant Ewing's Sarcoma Stem Cells That Retain Sensitivity to EWS-FLI1 Inhibition.. PLoS One.

[42] Dreux N, Marty M, Chibon F, Velasco V, Hostein I (2010). Value and limitation of immunohistochemical expression of HMGA2 in mesenchymal tumors: about a series of 1052 cases.. Mod Pathol.

[43] Henriksen J, Stabell M, Meza-Zepeda LA, Lauvrak SA, Kassem M (2010). Identification of target genes for wild type and truncated HMGA2 in mesenchymal stem-like cells.. BMC Cancer.

[44] Nishino J, Kim I, Chada K, Morrison SJ (2008). Hmga2 promotes neural stem cell self-renewal in young but not old mice by reducing p16Ink4a and p19Arf Expression.. Cell.

