# Supplementary figures and images for: Let-7a Is a Direct EWS-FLI-1 Target Implicated in Ewing's Sarcoma Development
Source: PLoS One. 2011 Aug 10;6(8):e23592. doi: 10.1371/journal.pone.0023592 (PMC3154507; doi:10.1371/journal.pone.0023592)

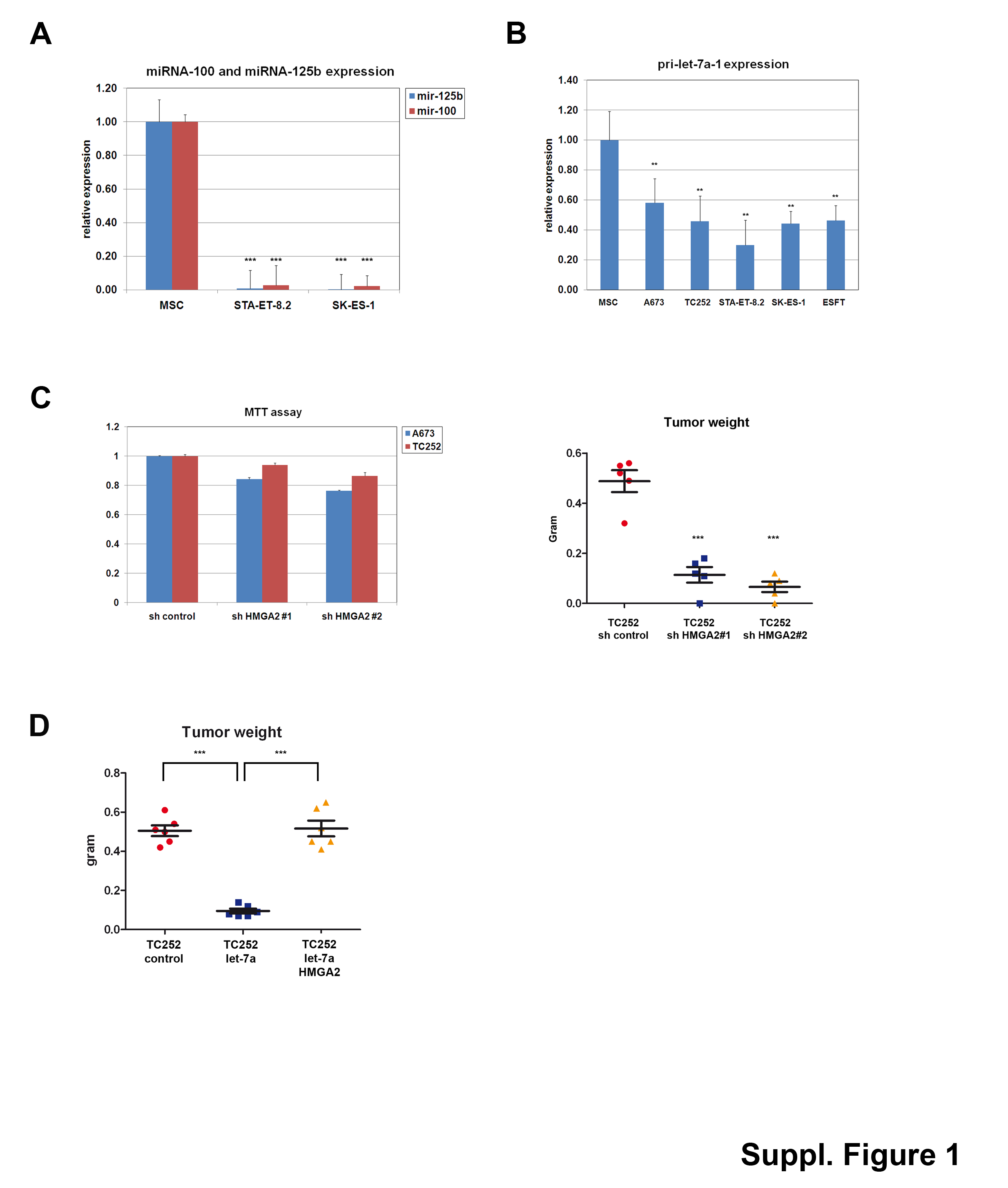

Supplement: Figure S1 — miRNA expression in STA-ET-8.2 and SK-ES-1 ESFT cell lines, MTT and tumorigenic assay of HMGA2-depleted TC252 tumor cells. A) Real-Time PCR analysis of miRNA-100, miRNA-125b in MSC, STA-ET-8.2 and SK-ES-1 cells. B) Real-Time PCR analysis of pri-let-7a-1 expression in MSC, ESFT cell lines and primary ESFT. C) Left: MTT assay of mock-infected and HMGA2-depleted ESFT tumor cells. Right: Depletion of HMGA2 in TC252 cells reduces corresponding tumor growth. D) HMGA2 expression in TC252 cells overexpressing let-7a rescues their tumorigenic properties. Error bars represent the SD of three independent determinations. Student T-test was used for statistical analysis, ** p<0.005, *** p<0.0005. (TIF) [file pone.0023592.s001.tif]

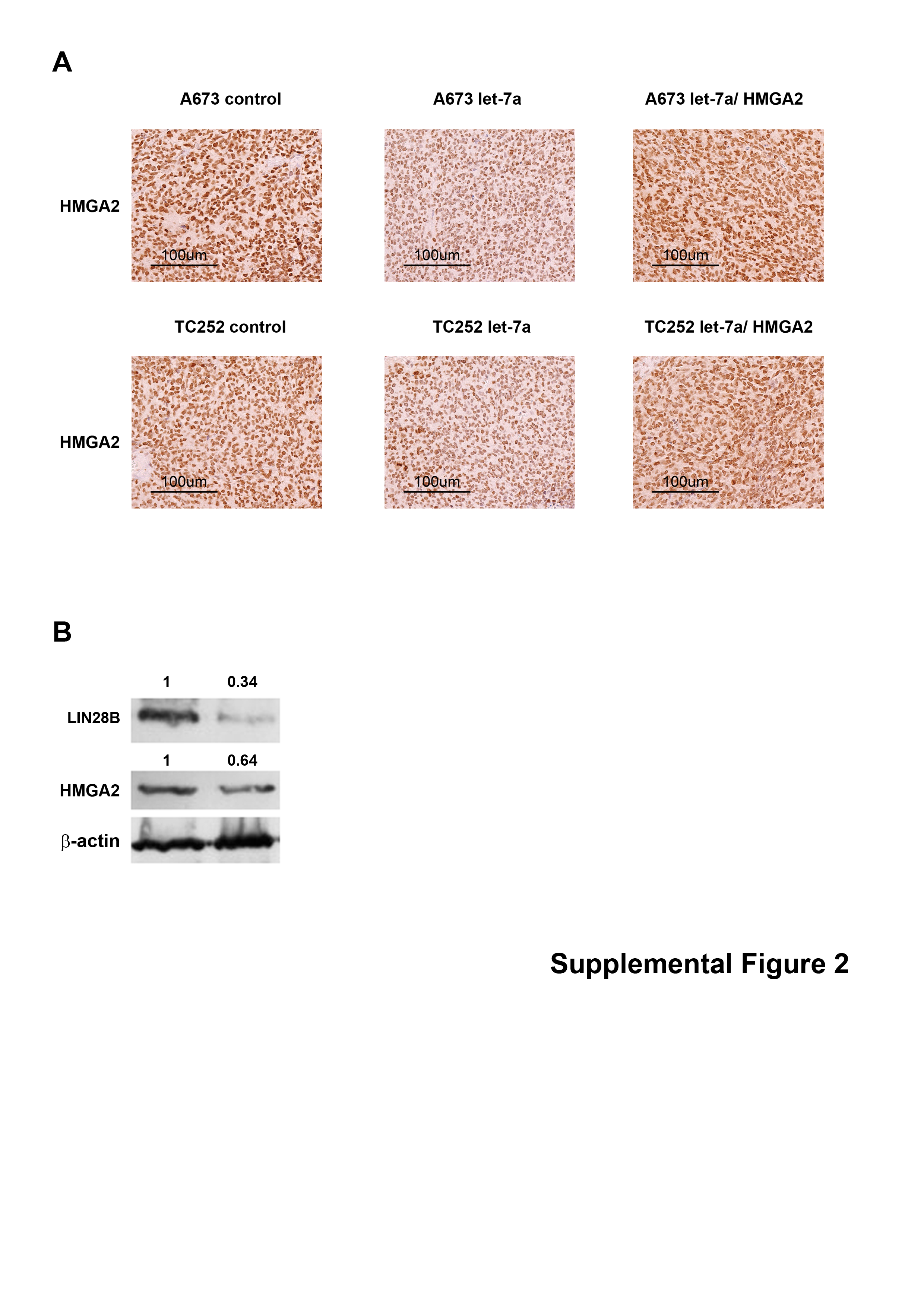

Supplement: Figure S2 — HMGA2 and Lin28 expression in let-7a and let-7a-HMGA2 expressing ESFT cells derived tumors. A) Immunohistochemical staining of HMGA2 in mock- let-7a and let-7a-HMGA2 expressing ESFT cell lines derived tumors. B) Western blot analysis of tumors from mice treated with let7a and vehicle only. Magnification 100x. (TIF) [file pone.0023592.s002.tif]
